# Allelic Expression Dynamics of Regulatory Factors During Embryogenic Callus Induction in ABB Banana (*Musa* spp. cv. Bengal, ABB Group)

**DOI:** 10.3390/plants14050761

**Published:** 2025-03-01

**Authors:** Xiaobing Zhao, Yiting Zhuang, Wangyang Xie, Yixin Yang, Jingyu Pu, Zhengyang Fan, Yukun Chen, Yuling Lin, Zhongxiong Lai

**Affiliations:** 1 Institute of Horticultural Biotechnology, Fujian Agriculture and Forestry University, Fuzhou 350002, China; zxb1344@126.com (X.Z.); ffzzyy789@163.com (Z.F.); cyk68@163.com (Y.C.); 2 Center for Genomics and Biotechnology, Fujian Provincial Key Laboratory of Haixia Applied Plant Systems Biology, Key Laboratory of Genetics, Breeding and Multiple Utilization of Crops, Ministry of Education, Fujian Agriculture and Forestry University, Fuzhou 350002, China; zhuang_yiting@163.com (Y.Z.); xwy971211@163.com (W.X.); yyx1231209@163.com (Y.Y.); pjy13990877535@126.com (J.P.)

**Keywords:** embryogenic callus, specific allele, transcriptional regulation, browning

## Abstract

The regulatory mechanisms underlying embryogenic callus (EC) formation in polyploid bananas remain unexplored, posing challenges for genetic transformation and biotechnological applications. Here, we conducted transcriptome sequencing on cultured explants, non-embryogenic callus, EC, and browning callus in the ABB cultivar ‘MJ’ (*Musa* spp. cv. Bengal). Our analysis of differentially expressed genes (DEGs) revealed significant enrichment in plant hormones, MAPK, and zeatin biosynthesis pathways. Notably, most genes in the MJ variety exhibited balanced expression of the A and B alleles, but A-specific allele expression was dominant in the key signaling pathways, whereas B-specific allele expression was very rare during EC induction. In the auxin signaling pathway, six A-specific *MJARF* genes were markedly downregulated, underscoring their critical roles in the negative regulation of callus formation. Additionally, six A-specific *MJEIN3* alleles were found to play negative regulatory roles in ethylene signaling during EC development. We also identified phenylpropanoids responsible for enzymatic browning. Furthermore, the expression patterns of transcription factors in bananas exhibited specific expression modes, highlighting the unique mechanisms of callus formation. This study enhanced our understanding of the regulatory roles of these alleles in EC induction and offers new insights into the utilization of alleles to improve the efficiency of somatic embryogenesis in bananas.

## 1. Introduction

Bananas (*Musa* spp.) are perennial, herbaceous, monocotyledonous plants belonging to the Musaceae family within the order Zingiberales. As one of the most produced fruits globally, bananas are a staple food for millions of people. The ABB group, which consists of starchy cooking bananas, plays a crucial role in food security, particularly in Southeast Asia [1]. Almost all cultivated banana varieties originated from the intra- and interspecific hybridization of two wild species in the Eumusa section, namely *Musa acuminata* (AA) and *Musa balbisiana* (BB), as well as long-term evolutionary variations, resulting in different genomic types such as diploids (AA, AB, and BB), triploids (AAA, AAB, ABB), and tetraploids (AAAA, AAAB, AABB, ABBB) [2]. Most commercial bananas are triploids, making it challenging to improve them using traditional breeding methods. To enhance the diversity, disease resistance, and cold tolerance of banana varieties, breeding bananas using biotechnological approaches is inevitable. The establishment of a robust genetic transformation receptor system is the foundation of plant genetic engineering [3]. A deeper understanding of the regulatory mechanisms governing banana embryogenic callus (EC) will significantly enhance genetic transformation efficiency.

The receptor system for exogenous genes in bananas is much more complex than the general tissue culture and reproduction systems used for banana production. Embryogenic cell suspension (ECS) lines have been successfully established in various banana varieties (lines) with multiple genotypes (AA, AB, AAB, ABB, AAA, and AAAA) [4,5]. The embryogenic system has been successfully used for Banana *Agrobacterium* transformation [6]; however, the transformation efficiency remains relatively low. Genotypes are the most crucial factor affecting efficiency. In this sense, somatic embryogenesis (SE) has been successfully achieved in several banana genotypes, including AA, BB, AB, AAA, ABB, and AAB) [3]. Currently, three varieties of *Musa* AAB, namely ‘French Plantain’, ‘Mysore’, and ‘Silk’, have been reported with SE induction efficiencies of 2%, 3%, and 7%, respectively. However, the *Musa* AAA ‘Grande Naine’ variety exhibited the highest induction rate among all the tested varieties, reaching 37% [7]. The reason for the highest induction rate in AAA varieties, and whether this was due to gene duplication in the A genome, has not yet been reported. Despite the existing research on SE induction in bananas, the process still faces challenges of low and unstable induction rates. The low induction efficiency of EC is still the key to hindering the genetic transformation of bananas [3]. Therefore, unveiling the key genes and molecular mechanisms underlying somatic embryogenic callus induction has become a focal point of research on the banana embryogenic regeneration system.

The use of plant genetic transformation systems to investigate gene functions has become a widely adopted research approach. Recent reports have suggested that the overexpression of certain key genes can significantly enhance the ability of plants to induce callus differentiation and regeneration, thereby improving the efficiency of genetic transformation. Overexpression of the maize *GOLDEN2* gene in rice and maize callus tissues promotes callus differentiation, consequently enhancing genetic transformation efficiency [8]. Similarly, the *TaWOX5* gene could significantly enhance wheat embryogenic calli and plant regeneration. Utilization of the *TaWOX5* gene markedly improves genetic transformation efficiency and reduces genotype dependence in most wheat varieties [9]. Furthermore, the *TaLAX1* gene enhances wheat malt regeneration by activating cytokinin synthesis and auxin transport-related gene expression, thereby enhancing wheat genetic transformation and gene-editing efficiency [10]. In *Arabidopsis*, ectopic expression of *LEC2* promotes SE, whereas, in cassava, *MeLEC2* overexpression induces embryogenesis, making it a valuable tool for transformation recovery [11,12]. Furthermore, *HB52* and *CRF3* overexpression in *Arabidopsis* aids callus formation without requiring exogenous auxins, whereas *DoLEA43* overexpression enhances callus induction, such as *WIND1* expression [13,14]. All these genes, which can significantly enhance callus differentiation and regeneration capabilities, have been successfully applied in genetic transformation following in-depth studies on the mechanisms of embryogenic calli and callus differentiation. This suggests that if we can identify the key genes involved in the induction of embryogenic calli in bananas and thoroughly elucidate their regulatory mechanisms, it may be possible to apply this knowledge to enhance the efficiency of banana genetic transformation.

There has been limited research on the regulatory mechanisms of EC induction and SE in bananas, and the main regulatory genes have not yet been identified. Understanding somatic cell development at the molecular level can be facilitated by transcriptome and proteome analyses [15]. Various differentially expressed proteins and genes involved in banana SE have been identified using multi-omics approaches, particularly transcriptomics and proteomics [16,17,18]. Studies suggest that embryogenic cells (ECs) are associated with an elevated accumulation of reactive oxygen species (ROS)-scavenging proteins, heat shock proteins (HSPs), and proteins involved in growth regulation [16,17]. Calcium signaling pathways and plant growth regulators, including indole-3-acetic acid (IAA), benzylaminopurine (BAP), and kinetin, play critical roles in the development and germination of banana somatic embryos [3].

The hormone signaling pathway, an important carrier of regulatory substances in organisms, plays an important role in callus formation [19]. Some studies have found that exogenous auxin can activate the expression of *LECs* and then activate the expression of auxin synthesis-related genes *YUC2* and *YUC4* and promote the development of *Arabidopsis* somatic embryo [20]. The LATERAL ORGAN BOUNDARIES DOMAIN (LBD)/asymmetric leaf 2-like transcription factor is involved in the regulation of callus formation in *Arabidopsis*. Auxin response factor ARF can activate the expression of the downstream LBD family of transcription factors LBD16, LBD17, LBD18, and LBD29 [21]. The molecular mechanism by which cytokinins promote callus formation remains to be further studied; however, B-ARRs, key transcription factors of the cytokinin signaling pathway, are closely related to callus formation [22]. Studies on *Arabidopsis thaliana* showed that overexpression of *ARR4* significantly promoted bud formation in the presence of cytokinin, while overexpression of *ARR8* inhibited bud formation and callus greening [23]. Overexpression of *ARR1* in a medium containing cytokinins promotes the formation of *Arabidopsis* calli [24]. Mitogen-activated protein kinase (MAPK) is an important signal transmitter from the cell surface to the nucleus and plays a crucial regulatory role in plant growth and development [25,26]. Transcriptome sequencing of different calli from two *Eucalyptus* species revealed that most of the genes were involved in signaling pathways such as plant hormones and MAPK [27]. In *Hevea brasiliensis*, differentially expressed genes (DEGs) were mainly enriched in MAPK and plant hormone signaling, biosynthesis, and metabolic pathways of secondary metabolites [28]. Numerous genes associated with SE have been identified through transcriptome analyses and callus induction, including ABA-induced genes [29], AGL 15 [30], calmodulin [31], DcAGP [32], LEC [33], and SERK [34]. Further studies suggest that the transcription factors BBM, WUS, VIVIVIPAROUS1, and LEC may play important roles in banana SE, particularly BBM2 and WUS2, in both embryogenic and non-embryogenic cell suspensions [18]. To date, the regulatory network for EC induction in triploid bananas of the ABB genome group has not been established, and the contributions of different alleles during the induction process remain unclear. The “Bengal Banana” (*Musa* spp. cv. Bengal, ABB Group, referred to as MJ banana) is part of the cooking banana series and was introduced from Bangladesh to China. Its primary phenotypic traits include a robust plant structure, large fruit size, and strong resistance to both diseases and drought [35]. Investigating the EC regulation mechanism of the MJ cultivar and establishing its callus induction system is crucial for developing transgenic banana varieties that are cold-tolerance, disease-resistant, and of high quality.

In this study, we developed an EC induction system for the MJ cultivar and uncovered a framework for the molecular events of callus formation in the ABB banana MJ. Furthermore, we elucidated the functional roles of the A and B alleles, providing a critical foundation for a deeper understanding of the regulatory mechanisms underlying EC formation in triploid bananas.

## 2. Results

### 2.1. Morphologies of Calli and the Crucial Role of the Growth Regulator 2,4-D in Promoting EC Induction

**Figure 1:**
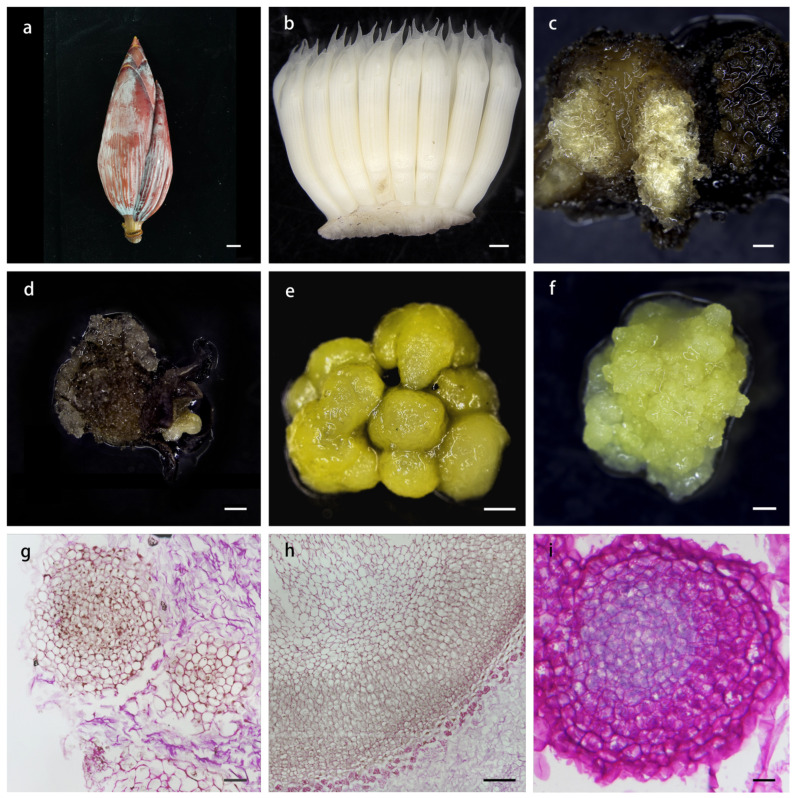
The process of embryogenic callus (EC) induction and morphological observation in banana MJ. (**a**) MJ male inflorescence. (**b**) The internal flower combs of MJ banana. (**c**,**d**) Crystalline small granular callus on B2 medium for 12 months. (**e**) Yellowish large granular callus cultivated on B2 medium for 8 months; (**f**) EC cultivated on B2 medium for 14 months. (**g**,**h**) Paraffin section of banana non-embryogenic callus as c and e, respectively. (**i**) Paraffin section of banana EC as (**f**). Scale bar = 2 cm (**a**), scale bar = 1 mm (**b**–**f**), scale bar = 20 μm (**g**–**i**).

When cultured on previously reported callus induction media with varying hormone ratios [36,37], the MJ cultivar (ABB genotype) exhibited the earliest initiation of callus growth. Therefore, we selected this cultivar as the primary experimental material. We developed several distinct culture media to induce callus formation and observed that immature flowers of the MJ cultivar could be induced to form callus on any of the four media tested (Supplementary Table S1). After a 5-month induction period, explants from immature male flowers showed the highest callus induction rate on B1 medium with 0.5 mg/L 2,4-D, resulting in the emergence of crystalline small granular callus on the explant surfaces (Figure 1a–d, Supplementary Table S1). The callus induction rate on the B2 medium with 1 mg/L 2,4-D was similar, with most explants producing larger yellowish granular calli (Figure 1e) and small crystalline granular calli. However, some explants on B2 medium produced velvety white calli. The induction rate on B3 medium with 2 mg/L 2,4-D was significantly decreased compared to that on B1 and B2, showing a mix of crystalline small granular calli and yellowish larger granular calli (Supplementary Table S1). For the B4 medium with 4 mg/L 2,4-D, the induction rate of the explants was 28.1% (Supplementary Table S1). The callus state was suboptimal, with a low number of induced calli, exhibiting velvety white calli and very few crystalline small granular calli with a more pronounced brown coloration. From these results, we concluded that the concentration of 2,4-D is crucial for callus induction rate and quality. Embryogenic calli of Banana MJ were successfully induced from the B2 medium after 9 months of induction, displaying a soft, yellow, moist, and loose morphology (Figure 1f). Therefore, the B2 medium (basal medium supplemented with 1 mg/L 2,4-D, 1 mg/L NAA, and 1 mg/L IAA) was determined to be the optimal medium for subsequent experiments.

To further analyze the morphology of callus cells at different developmental stages, we examined the status of various callus cell forms in paraffin sections. The non-embryogenic calli appeared white, loose and lacked embryo-like structures on the surface (Figure 1g). The cells of the non-embryogenic calli were highly vacuolated, contained fewer starch granules, and exhibited abnormal shapes and sizes (Figure 1g–h). Larger yellowish granular calli were another special non-embryogenic callus (Figure 1e), suggesting that they may be intermediates in the process of EC formation or SE. In contrast, the embryogenic calli of MJ were pale yellow and delicate. These cells were small, spherical, and had centrally located nuclei. Both the nucleus and the cytoplasm were deeply stained and displayed characteristics typical of ECs. Darkly stained cell aggregates confirmed the presence of dense cytoplasm with abundant starch granules in the EC (Figure 1i). Embryogenic cells were spherical and multiplied at a high rate.

### 2.2. Comparison of Transcriptional Profiling During Formation of EC in ABB Banana

**Figure 2:**
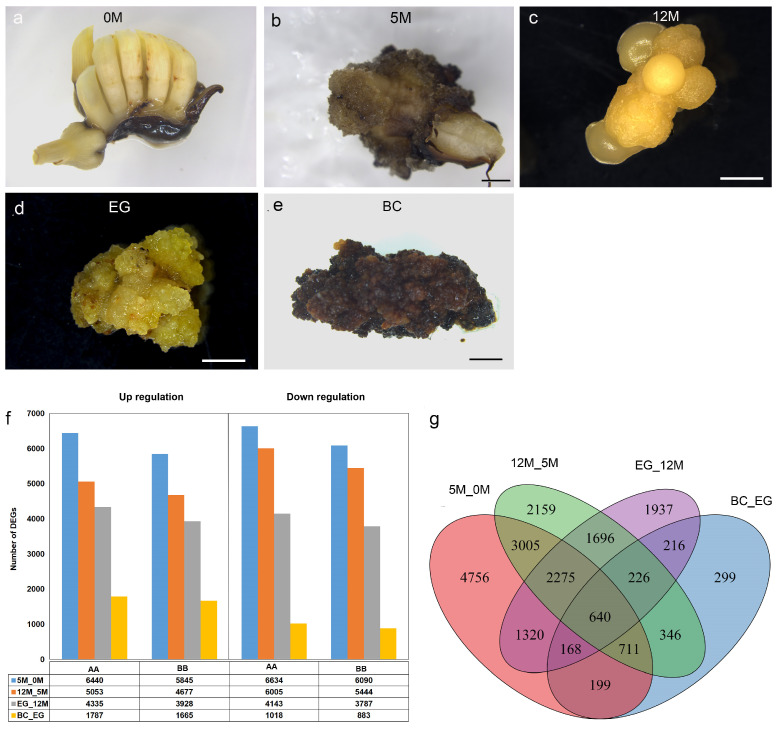
Overview of differentially expressed genes among five samples during the stages of EC induction in banana MJ. (**a**) MJ flower buds. (**b**) Explants induced on B2 medium for 5 months (5M). (**c**) Explants induced on B2 medium for 12 months (12 M). (**d**) EC obtained after 14 months of induction on the B2 medium. (**e**) Browned EC (BC). (**f**) The number of DEGs up- or downregulated during embryogenic callus formation. (**g**) Venn diagram showing overlap and specific DEGs in five samples. Scale bar = 2 mm (**a**–**e**).

To identify the key DEGs and essential pathways involved during banana EC induction, we selected samples from the five stages for global transcriptome analysis. These stages included immature male flowers of bananas cultivated on B2 medium for one week (0M), flowers cultivated on B2 medium for 5 months (5M) and 12-month callus (12M), embryogenic calli (EC), and browning embryogenic calli (BC) (Figure 2a–e). The published genome of *M. acuminata* (AA genome) of DH-Pahang and *Musa balbisiana* (BB genome) of DH-PKW were used as reference genomes for transcriptome data analysis [38,39]. The correlation between the biological replicates for each stage was calculated based on the FPKM results. Pearson’s correlation analysis indicated a high linear correlation among different samples (Supplementary Figure S1a). Furthermore, the PCA of the 15 samples (with three biological replicates for each sample) showed clear distinctions at different stages and a compact distribution of biological replicates (Supplementary Figure S1b), indicating that the data quality was highly reproducible, allowing for further analysis.

We identified 6440, 5053, 4335, and 1787 upregulated DEGs and 6634, 6005, 4143, and 1018 downregulated DEGs across four comparisons: 5M versus 0M, 12M versus 5M, EC versus 12 M, and BC versus EC, respectively, using the AA genome as a reference (Figure 2f–g). Similar patterns of upregulated and downregulated DEGs were observed when the BB genome was used as a reference (Figure 2f). Additionally, using the AA genome as a reference, we identified 4756, 2159, 1937, and 299 specific DEGs across the four groups, respectively (Figure 2g). Similarly, using the BB genome as a reference, we detected 4286, 2026, 1696, and 256 DEGs in the respective groups (Supplementary Figure S2). The number of specifically expressed genes progressively decreased across the four stages, suggesting that gene expression was significantly influenced during the early phases of explant differentiation. To investigate the specific expression of the A and B alleles in MJ bananas further, we identified homologous and specific genes using scripts based on mapping of the AA and BB genomes. We identified 28,969 homologous genes, 7936 A-specific genes, and 2772 B-specific genes in the MJ bananas. The number of A-specific genes was 2.8 times greater than that of B-specific genes, indicating the significant predominance of A-specific genes in the MJ ABB genome.

### 2.3. Global Analysis of DEGs Expressed in Calli During the EC Induction of ABB Banana

Subsequently, GO classification and KEGG pathway analyses were conducted for the DEGs across the four comparisons. In the comparison between the 0M and 5M callus, DEGs were significantly enriched in biological processes such as response to stimulus, response to stress, embryogenic root development, and protein phosphorylation (Supplementary Figure S3a).

Additionally, in the comparison between the 12M and 5M callus, DEGs were enriched in biological processes, including response to stimulus, response to stress, oxidation–reduction, and DNA metabolic processes (Supplementary Figure S3b). As the 12M callus developed into an EC, the DEGs were primarily enriched in plant organ development, response to hormones, response to organic substances, and response to acidic chemicals (Supplementary Figure S3c). When comparing browning calli and embryogenic calli, enrichment was observed for phenylpropanoid metabolic processes, phenylpropanoid biosynthetic processes, secondary metabolic processes, lignin metabolic processes, and flavonoid metabolic processes (Supplementary Figure S3d).

Furthermore, significant differences were identified in the KEGG pathways of DEGs during the formation of embryogenic calli in MJ at various stages. In the comparison between 12M and 5M callus, DEGs were enriched in pathways such as plant hormone signal transduction, tyrosine metabolism, fatty acid degradation, ABC transporters, and starch and sucrose metabolism (Supplementary Figure S4a). Following the development of the 5-month callus, the enriched pathways included plant hormone signal transduction, phenylpropanoid biosynthesis, glutathione metabolism, starch, and sucrose metabolism, and the MAPK signaling pathway (Supplementary Figure S4b). Notably, aside from metabolism and secondary metabolism, the hormone signaling pathway emerged as the most significantly enriched pathway, underscoring the pivotal role of hormones at this stage. Additionally, KEGG enrichment analysis revealed a distinct MAPK signaling pathway in plants. Previous evidence suggests that MAPK plays a crucial role in physiological processes such as the regulation of plant growth, development, and stress resistance [40].

In the comparison between 12M callus and embryogenic calli, plant hormone signal transduction and MAPK signaling pathways were the two most enriched pathways. Concurrently, significant enrichment was observed in metabolites such as glutathione metabolism, tropane, piperidine, and pyridine alkaloid biosynthesis; phenylpropanoid biosynthesis; and starch and sucrose metabolism (Supplementary Figure S4c). When comparing browning calli to normal calli, pathways including phenylpropanoid biosynthesis, tropane, piperidine, and pyridine alkaloid biosynthesis, phenylalanine, tyrosine, and tryptophan biosynthesis, glutathione metabolism, and flavonoid biosynthesis were predominantly enriched (Supplementary Figure S4d), suggesting that the accumulation of metabolites primarily begins at the EC stage. Subsequently, we focused on the changes in the expression levels of genes significantly enriched in these pathways to elucidate the cellular changes occurring in plant cells.

**Figure 3:**
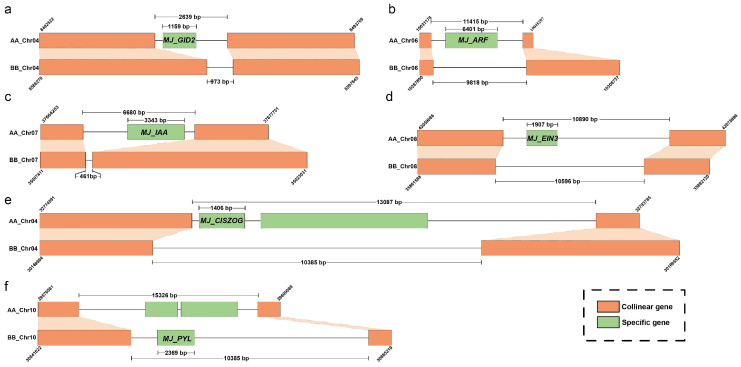
Visualization of the Specific Gene Locations Identified in the AA and BB Genomes. Specific genes within the AA and BB genomes were identified using BLASTn, and the positions of homologous genes flanking these specific genes in both genomes are displayed. This figure demonstrates the accuracy of specific gene identification and indicates that these specific genes arise due to insertion and deletion mutations. (**a**–**d**) Panels illustrate specific genes in the hormone signaling pathways, including *Macma4_04_g11940 (MJ_GID2)*, *Macma4_06_g14450 (MJ_ ARF)*, *Macma4_07_g28170 (MJ_IAA)* in the A genome compared to the B genome, *Macma4_02_g00180 (MJ_CALM)* in the MAPK signaling pathway, *Macma4_08_g22960 (MJ_EIN3)*, and *Macma4_04_g24700 (MJ_CISZOG)* in the zeatin biosynthesis pathway (**e**), as well as the specific gene *Mba10_g12780 (MJ_PYL)* in the BB genome (**f**).

Next, we concentrated on the plant hormone signaling pathways, MAPK signaling pathways, and zeatin biosynthesis signaling pathways to uncover the critical roles of A-specific and B-specific alleles in the banana MJ EC induction process. Consequently, we identified 48, 32, and 5 genes within the A-specific alleles in three signaling pathways. Interestingly, we identified only two specific B alleles expressed in the hormone signaling pathway, whereas no specific B allele was identified in the MAPK or zeatin biosynthesis signaling pathways (Supplementary Tables S2 and S3). We selected seven specific genes for identify chromosomal position and specificity determination, encompassing six A-specific genes: *Macma4_04_g11940 (MJ_GID2)*, *Macma4_06_g14450 (MJ_ARF)*, *Macma4_07_g28170 (MJ_IAA)*, *Macma4_02_g00180 (MJ_CALM)* involved in the MAPK signaling pathway, *Macma4_08_g22960 (MJ_EIN3)*, and *Macma4_04_g24700 (MJ_CISZOG)* associated with the zeatin biosynthesis pathway, in addition to one B-specific gene, *Mba10_g12780 (MJ_PYL)*. Through a comprehensive synteny analysis of the genes flanking these seven specific loci, we observed that the interval lengths of these A-specific genes and their adjacent syntenic genes were significantly greater than those of the syntenic genes in the BB genome (Figure 3). The results showed that the emergence of these specific genes was attributable to gene loss in the BB genome or insertional structural variations. This finding not only corroborates the precision of identifying specific genes but also reveals potential mechanisms underlying the formation of these specific genes.

### 2.4. The Dominant Expression Changes of Genes Involved in Auxin Signaling During EC Formation

To elucidate the regulatory network involved in EC dedifferentiation, we analyzed the DEGs enriched in hormonal signaling pathways across the four stages of callus development. Specifically, 234, 210, 183, and 58 genes were enriched in the comparisons of 5M vs. 0M, 12M vs. 5M, EC vs. 12M, and BC vs. EC, respectively. A Venn diagram was constructed to identify the stage-specific genes (Figure 2g, Supplementary Figure S2), highlighting the significant involvement of hormone-related genes in the differentiation potential of embryogenic calli. Our initial focus was on the auxin and cytokinin signaling pathways during callus induction.

**Figure 4:**
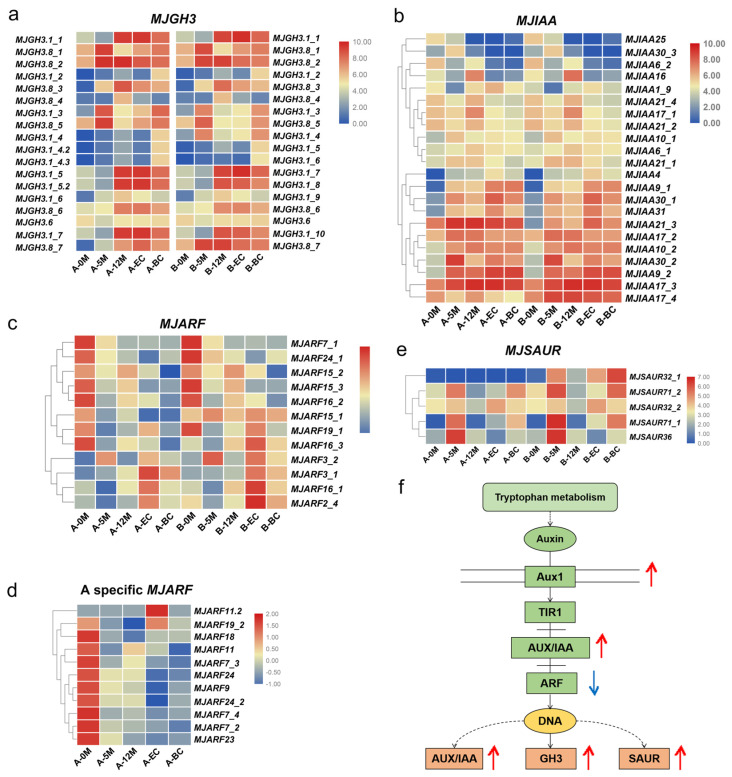
Expression of genes involved in the auxin pathway during embryogenic callus formation. The expression levels were visualized by using OmicStudio tools at https://www.omicstudio.cn/tool (accessed on 5 December 2023). Numbers beneath the heat map indicate the relative expression intensities and the higher expression intensities are indicated by more reddish colors. Identification of A and B alleles in the ABB genome was conducted using allele tables, where A-5M denotes the gene expression levels of the A allele at the 5M stage, and B-5M denotes those of the B allele at the same stage. (**a**–**c**,**e**) The expression levels of *MJGH3s*, *MJIAAs*, *MJARFs*, and *MJSAUR*, which are associated with the auxin pathway. (**d**) A heat map showing A-specific allele expression levels of *MJARF*. (**f**) The diagram illustrates the upregulation and downregulation of components in the auxin signaling pathway.

The primary inactivation route for the natural auxin indole-3-acetic acid (IAA) involves the GH3-ILR1-DAO pathway. Initially, GH3 IAA amidosynthetases transform IAA into IAA-amino acid conjugates. These conjugates, specifically IAA-aspartate (IAA-Asp) and IAA-glutamate (IAA-Glu) act as storage forms of IAA. They can be reconverted to IAA through the action of ILR1/ILL amidohydrolases [41]. We identified 17 A allele GH3 genes and 19 B allele GH3 genes. Among the A alleles, *MJGH3.8_1*, *MJGH3.8_2*, *MJGH3.1_3*, and *MJGH3.8_5* were significantly upregulated during the 5M stage of MJ, whereas *MJGH3.1_1*, *MJGH3.1_5*, and *MJGH3.8_7* showed notable induction at the 12M stage, with a marked increase in expression in the EC (Figure 4a). These findings suggest that following callus induction, *MJGH3* expression is induced by auxin in the culture medium, leading to the production of more IAA-amino acid conjugates within the cells. This indicated that GH3 plays a crucial role in maintaining auxin concentrations and ensuring cell division, thereby promoting callus induction.

In the auxin signaling pathway, Aux/IAA plays an inhibitory role in the regulation of auxin-responsive gene expression. Aux/IAA proteins form dimers with the Auxin Response Factors (ARFs), thereby inhibiting their transcriptional regulatory functions of ARFs. ARF transcription factors can bind to the cis-regulatory elements of auxin-inducible genes and regulate their transcriptional expression [42]. We identified 27 differentially expressed members of the IAA family in both A and B alleles during MJ callus induction. Notably, *MJIAA21_2*, *MJIAA30_3*, *MJIAA16*, and *MJIAA21_4* were notably induced at the 5M stage of callus initiation, with B alleles showing a consistent expression pattern (Figure 4b). This suggests that IAA transcriptional repressors were significantly upregulated after callus induction, initiating transcriptional repression. Additionally, 12 differentially expressed ARF genes were identified. Interestingly, these alleles exhibited low expression levels during the MJ callus induction stage, except for one gene, *MJARF24_1*, which was highly expressed in MJ explants but showed a significant decrease in expression at the 5M stage (Figure 4c,f). Notably, we identified 11 specific A alleles of *MJARF* genes, all of which displayed a significant downward trend during callus induction (Figure 4d,f). These findings indicate that, during banana callus induction, the accumulation of IAA family members leads to an increase in bound ARFs, resulting in decreased ARF expression, thereby regulating callus proliferation and differentiation.

Small auxin-up RNA (SAUR) is a rapidly responsive gene to auxin [43]. A total of five *MJSAUR* genes that exhibited differential expression during the callus induction process with MJ treatment were identified. Notably, almost all these genes showed a significant increase in expression at the 5M stage (Figure 4e,f). This suggests that *MJSAUR* plays a crucial role in the early response to callus induction.

Cytokinin signaling relies primarily on downstream auxin response regulators (ARRs) to convey signals. Interestingly, we observed minimal differential expressions of cytokinin-related genes during callus induction in bananas. Only three ARR-B genes were identified as A-specific alleles. However, they exhibited very low expression levels in both MJ explants and callus induction stages (Supplementary Table S2). Additionally, cytokinin dehydrogenase (CKX) inactivates cytokinins by oxidatively removing their active side chains, rendering them biologically inactive. We identified one specific A allele of *MJCKX* with no differential expression; the B allele was not expressed during callus formation (Supplementary Tables S2 and S3).

### 2.5. BSK and BZR1 Are Involved in the Regulation of EC Induction

**Figure 5:**
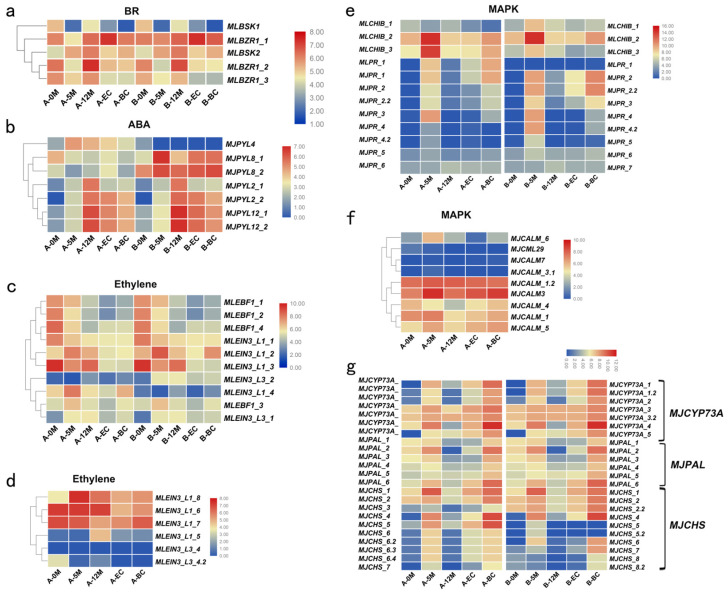
Expression of genes involved in the hormone pathway pathway during embryogenic callus formation. Numbers beneath the heat map indicate the relative expression intensities and the higher expression intensities are indicated by more reddish colors. Identification of A and B alleles in the ABB genome was conducted using allele tables, where A-5M denotes the gene expression levels of the A allele at the 5M stage, and B-5M denotes those of the B allele at the same stage. (**a**) A heat map illustrating gene expression levels in the BR signaling pathway. (**b**) A heat map for gene expression levels in the ABA signaling pathway. (**c**) A heat map depicting gene expression levels in the ethylene signaling pathway. (**d**) A heat map showing A-specific allele expression levels within the ethylene signaling pathway. (**e**) A heat map of gene expression levels in the MAPK signaling pathway. (**f**) A heat map of A-specific allele gene expression levels in the MAPK signaling pathway. (**g**) The expression levels of *MJCYP73As*, *MJPALs*, and *MJCHSs*, which are associated with embryogenic callus browning.

Brassinosteroids (BRs) constitute a class of plant steroid hormones playing pivotal roles in plant growth and development [44,45]. Studies have demonstrated that supplementation with BRs at suitable concentrations enhances SE formation in various plant species, including plumules [46], *Coffea arabica* [47], and *cotton* [48]. In our study, we identified five DEGs enriched in brassinosteroid (BR) synthesis and signal transduction pathways, comprising two BR-signaling kinase genes (BSK) and three brassinosteroid-resistant 1/2 genes (BZR1_2). The expression abundance of the *MJBSK1* gene peaked during the 0M period, followed by initial downregulation and subsequent gradual upregulation after the 12M period. Conversely, the expression of the *MJBSK2* gene was upregulated during the 5M period, reaching its peak at the 12M period and then gradually decreasing during EC development, with an interesting upregulation observed in the browning EC (Figure 5a). For the *MJBZR1_1* genes, expression abundance peaked in the EC, whereas *MJBZR1_2* expression peaked at the 12M stage (Figure 5a). The expression patterns of the B allele of *MJBSK1* and *MJBZR1* were highly similar to those of the A allele, suggesting the conservation of *BSK* and *BZR1* in the functions of both A and B alleles (Figure 5a). These results indicated that *MJBZR1_1* and *MJBZR1_2* play positive roles in inducing EC formation.

### 2.6. Abscisic Acid Pathway Gene PYL Is Very Important to Respond EC Induction

Abscisic acid (ABA) is a classical plant growth inhibitor that regulates various physiological processes, including embryo maturation, seed dormancy, germination, cell division, and elongation [49]. ABA also inhibits the germination of mature embryos and their conversion into plantlets [50]. ABA is perceived by the intracellular PYR/PYL/RCAR receptors, which are encoded by *PYL* genes [51,52]. When ABA binds to PYL receptors, it triggers a signaling cascade that activates downstream responses [53]. In our study, we identified eight DEGs enriched in the abscisic acid receptor PYR/PYL family. Four *MJPYL* genes (*MJPYL2_1*, *MJPYL12_1*, *MJPYL12_2*, and *MJPYL2_2*) were highly expressed during EC induction. However, the B alleles of *MJPYL8_1* and *MJPYL8_2* were significantly upregulated in Stage 2 (five months after callus induction in explants), whereas the expression of the A alleles showed no significant changes (Figure 5b). This indicated that the B alleles of these two alleles were specifically expressed. Interestingly, we also identified one B allele of *MJPYL* that was specifically expressed and exhibited the highest expression levels in the embryogenic calli (Supplementary Table S3). This suggests that the B-specific allele of *MJPYL* is crucial for EC formation in MJ bananas.

### 2.7. Genes Involved in Ethylene Signaling Negatively Regulate the Formation of EC

Ethylene regulates organogenesis and SE, and its role has been extensively studied [54]. EBF1 (EIN3-Binding F-box 1) and EIN3 (Ethylene Insensitive 3) are key components of the ethylene signaling pathway, where EBF1 negatively regulates EIN3 by promoting its degradation. In our study, three out of four *MJEBF1* genes showed high expression in the explants, but all were downregulated after callus induction, with similar trends observed for both the A and B alleles (Figure 5c). Furthermore, six *MJEIN3* genes exhibited similar expression patterns in both the A and B alleles. Among them, *MJEIN3_L1_1* and *MJEIN3_L1_3* showed the highest expression in the explants, with their expression levels gradually decreasing after callus induction. However, *MJEIN3_L1_2* and *MJEIN3_L1_4* displayed different patterns, with their expression levels significantly upregulated at Stage 2 (five months after callus induction), followed by a decrease (Figure 5c). Interestingly, we identified six *MJEIN3* genes that were specifically expressed in the A allele. Among these, *MJEIN3_L1_5, MJEIN3_L3_4,* and *MJEIN3_L3_4.2* exhibited very low expression levels, whereas *MJEIN3_L1_6, MJEIN3_L1_7*, and *MJEIN3_L1_8* exhibited allele-specific expression (Figure 5d). Specifically, *MJEIN3_L1_6* and *MJEIN3_L1_7* alleles negatively regulated callus formation, whereas *MJEIN3_L1_8* was significantly highly expressed only at stage 2, and its expression decreased at other stages (Figure 5d). These findings indicate that *MJEBF1* and *MJEIN3* serve as negative regulators of callus induction. Notably, the expression of specific *MJEIN3* A alleles appears to play a crucial role in negative regulation during embryogenic development. This suggests a complex regulatory mechanism by which these alleles modulate key developmental processes, potentially influencing the efficiency and outcomes of callus formation.

### 2.8. Specific A Alleles Involved in the MAPK Signaling Pathway Positively Regulates the Development of EC

In plants, MAPK signaling pathways play crucial regulatory roles in various biological processes, including responses to biotic and abiotic stresses, as well as hormone and developmental signaling pathways [55]. BraMAPK3 demonstrates a rapid response to treatments involving salt, heat, waterlogging, wounding, 6-BA, and NAA, suggesting that BraMAPK3 may be a key regulator of abiotic stress responses [56]. Additionally, miRNAs target genes within the MAPK signaling pathway to regulate the accumulation of functional metabolites in longans [57].

We investigated the MAPK signaling pathway and identified 128 and 103 DEGs in comparisons between 12M vs. 5M and between EC vs. 12M, respectively. Among these, 68 DEGs were common to both comparisons, whereas 60 of 128 and 35 of 103 DEGs were unique to each comparison. CHIB basic endochitinase B (CHIB) and pathogenesis-related protein 1(PR1) are both involved in plant defense mechanisms and can be linked to the MAPK signaling pathway, which is crucial for transmitting stress and defense signals in plants [58]. Specifically, we identified three *CHIB*, six *PR1* for the A allele, and three *CHIB* and seven *PR1* genes for the B allele that were differentially expressed during callus formation in Banana MJ (Figure 5e).

Notably, the three *CHIB* genes for the A and B alleles were significantly upregulated at the 5-month callus stage but were downregulated at other stages, particularly *MJCHIB2* and *MJCHIB3*, indicating their dominance in banana callus formation. Plant pathogenesis-related (PR) proteins are crucial components of plant defense mechanisms and are primarily associated with responses to biotic and abiotic stress [59,60]. For the A allele of PR1, all *MJPR* were highly expressed in the 5-month callus and browning calli, except for the B alleles of *MJPR_1* and *MJPR_6* (Figure 5e). This indicates that the *MJPR1* gene exhibits a similar effect in both the A and B alleles.

Interestingly, our analysis revealed 32 genes associated with the MAPK signaling pathway within specific A alleles, including notable genes such as calmodulin (CALM), *MKK4_5*, *MKK9*, *MPK1*, and transcription factor MYC2 (Supplementary Table S2). Among these nine members of the *MJCALM* gene family, *MJCALM_1*, *MJCALM_1.2*, and *MJCALM3* (Figure 5f) showed remarkably high expression levels during callus formation. Of special interest, *MJCALM3* was significantly induced at the 5M stage, underscoring its pivotal role in callus induction. These observations imply that the 32 genes involved in the MAPK signaling pathway may have a substantial impact on callus induction.

### 2.9. Pheylalanine and Flavonoids Are the Main Phenolic Compounds Responsible for Enzymatic Browning

Tissue culture is an effective method for rapidly breeding seedlings and enhancing production efficiency. However, explant browning poses a key limitation to successful tissue culture. Enzymatic browning is the primary cause of explant browning in plant tissue culture. In banana tissue culture, browning of embryogenic calli is a significant hurdle in banana callus transformation. Through a comparative analysis between embryogenic calli and browning calli, we identified 49 DEGs significantly enriched in phenylpropanoid biosynthesis, 18 DEGs in the Tropane, piperidine, and pyridine alkaloid biosynthesis pathways, 17 DEGs in ubiquinone and other terpenoid–quinone biosynthesis pathway and 21DEGs in the Flavonoid Biosynthesis pathway. Cinnamate 4-hydroxylase (C4H; CYP73A) is a cytochrome P450 monooxygenase located in the endoplasmic reticulum of plants. This enzyme utilizes NADPH-cytochrome P450 reductase as an electron donor and hydroxylates cinnamic acid to produce 4-coumaric acid during phenylpropanoid metabolism. Notably, all six *MaCYP73A* genes and their alleles exhibited markedly upregulated expression levels in the browning calli, with particular emphasis on *MJCYP73A_5* (Figure 5g). In the phenylalanine metabolism pathway, genes encoding phenylalanine ammonia-lyase (PAL), peroxidase, and cinnamyl-alcohol dehydrogenase (CAD) displayed lower expression during the formation stage of the EC but significantly higher expression in browning calli. Notably, the inhibition of PAL activity has been linked to a reduction in phenolic compound biosynthesis, and our analysis identified six *MaPAL* genes and alleles that showed elevated expression in the browning callus compared to the EC (Figure 5g). CHS (chalcone synthase) is a key enzyme in the biosynthetic pathway of flavonoids. We identified nine MaCHS DEGs, four of which were specific genes lacking the B allele. All of these CHS genes exhibited elevated expression levels in the browning callus, with particular emphasis on the specific gene *MJCHS_5* (Figure 5g). When explants are cut, the cells are damaged, leading to the release of phenols that undergo enzymatic oxidation to form quinones, resulting in enzymatic browning [61].

### 2.10. Specific Expression of Transcription Factors During the EC Formation Process

**Figure 6:**
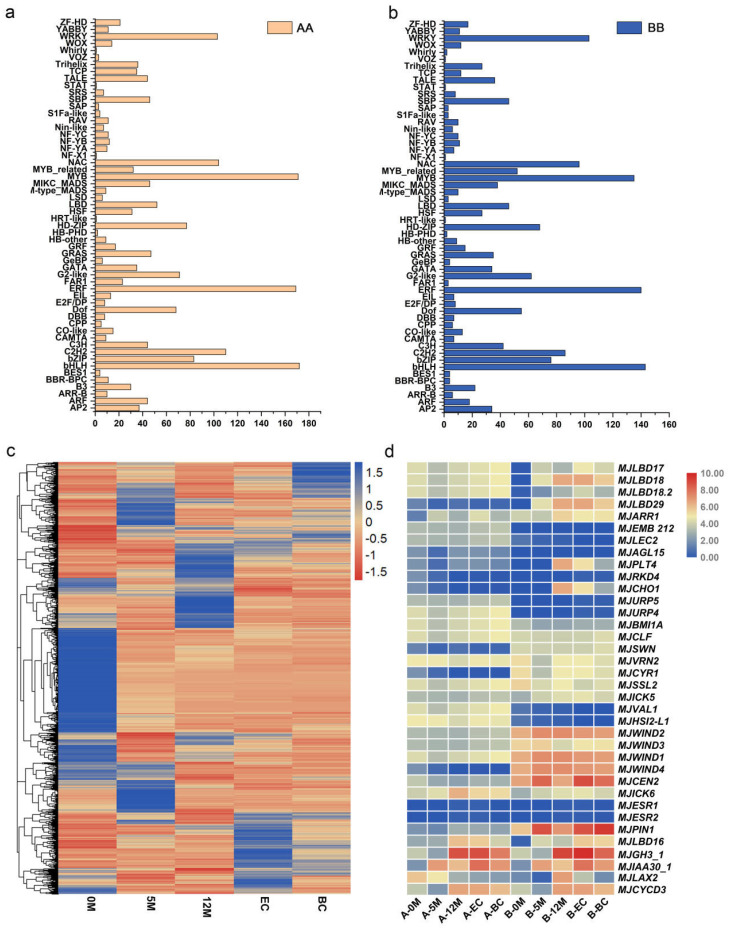
Expression levels of differentially expressed transcription factors and homologous genes previously reported to be involved in callus induction. (**a**,**b**) Statistics on the number of transcription factors encoded by differentially expressed genes identified during the banana embryogenic callus induction process, using AA and BB as reference genomes, respectively. (**c**) Expression levels of transcription factors encoded by differentially expressed genes during the embryogenic callus induction process using AA as reference. (**d**) Expression levels of homologous genes in banana MJ corresponding to genes reported in *Arabidopsis*, which are essential in the callus induction process. Genes highlighted in blue indicate lower expression levels of transcription factors (TFs), while those highlighted in red represent TFs with higher expression levels.

Transcription factors (TFs) are master regulators that play crucial roles in various biological processes by modulating a wide array of downstream targets. Our RNA-seq data revealed that the bHLH, MYB, ERF, NAC, and WRKY families were the top five TF families identified in the DEG involved in the four comparisons (Figure 6a,b). Notably, one-third of the transcription factors were significantly downregulated upon initiation of EC induction, indicating their negative regulatory role in this process. In contrast, the remaining two-thirds of the transcription factors exhibited varying degrees of upregulation during both the 5-month and 12-month stages of EC development, suggesting at positive regulatory function for the induction of embryogenic calli (Figure 6c).

**Figure 7:**
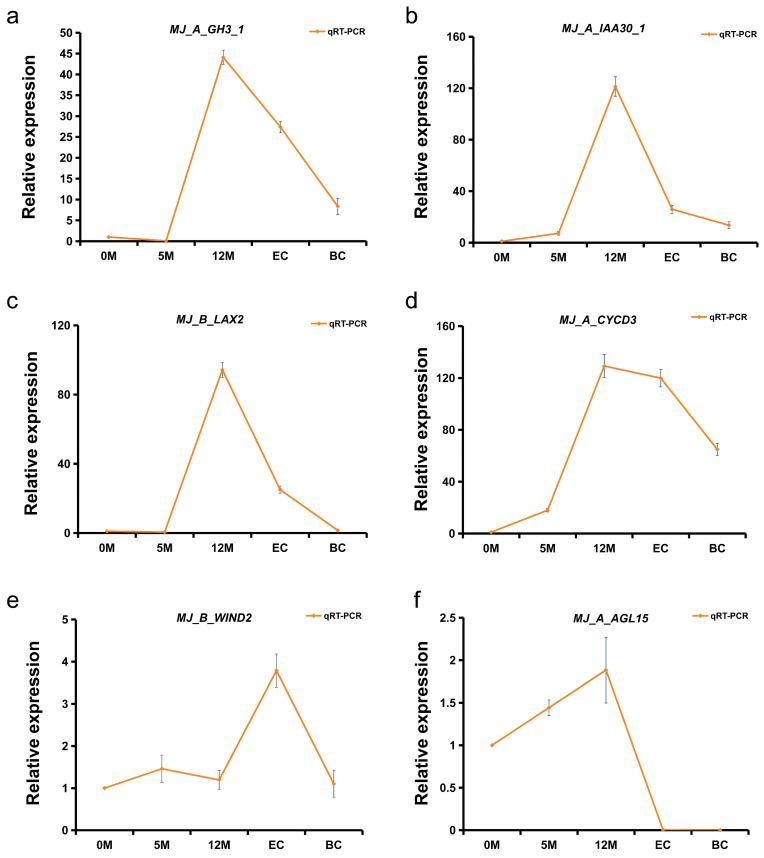
Transcript levels of four genes as determined by qRT-PCR. (**a**–**f**) Illustrate the expression of the A allele of *MJGH3* (*MJ_A_GH3.1*), *MJ_A_IAA30_1*, *MJ_A_CYCD3,* the B allele of *MJLAX2* (*MJ_B_LAX2*) and *MJ_B_WIND2*, respectively. The data presented are the means ± standard deviation (S.D.) from three biological replicates.

Over the past decade, studies of mutants with abnormal callus induction and development have revealed that callus induction is governed by intricate regulatory mechanisms [62]. Several key molecules involved in callus development regulation have been identified in *Arabidopsis*, many of which are crucial transcription factors. We investigated homologous genes in bananas and examined the expression of their A and B alleles. Interestingly, we found that nearly 80% of these homologous genes exhibited very low or no significant expression during EC induction in bananas. However, some previously reported transcription factors, such as *MJLBD* and *MJWIND1*, were significantly induced. Specifically, *MJLBD17* and *MJLBD18* were markedly induced in the BB genome, whereas both A and B alleles of the *MJLBD16* gene are induced during banana development. Additionally, *MJWIND1*, *MJWIND2*, *MJWIND3*, and *MJWIND4*, and the auxin transport carrier genes *MJPIN* and *MJLAX2* showed significant induction only in the B allele during EC induction. *CYCD3*, previously reported as a cell cycle-related gene, also showed significantly high expression of both the A and B alleles during the callus induction stage in bananas (Figure 6d). To validate the accuracy of the transcriptome data, we selected six genes (*MJGH3_1*, *MJIAA30_1*, *MHLAX2*, *MJCYCD3, MJWIND2, MJAGL15*) for quantitative analyses. The observed expression patterns were consistent with those of the RNA-Seq, confirming the reliability of the data (Figure 7, Supplementary Table S4). These findings suggest that the expression of these genes, which are known to play critical roles in callus induction in *Arabidopsis*, plays an allele-specific role in bananas.

## 3. Discussion

The induction of embryogenic calli in bananas is challenging. In this study, we successfully induced embryogenic calli in the ABB genotype of the banana MJ variety under various test conditions, and histological analysis confirmed the excellent state of the EC. Through transcriptome analysis during the formation of embryogenic calli, we discovered that auxin signaling pathway genes played a dominant role in the induction of MJ embryogenic calli, followed by the involvement of the MAPK signaling pathway, ABA, and ethylene. This indicates that the induction of banana callus is a process of coordinated hormonal signaling regulation, with callus induction and explant defense responses being synergistically controlled. Surprisingly, we demonstrated the minimal involvement of cytokinin signaling molecules in the establishment of embryogenic calli.

**Figure 8:**
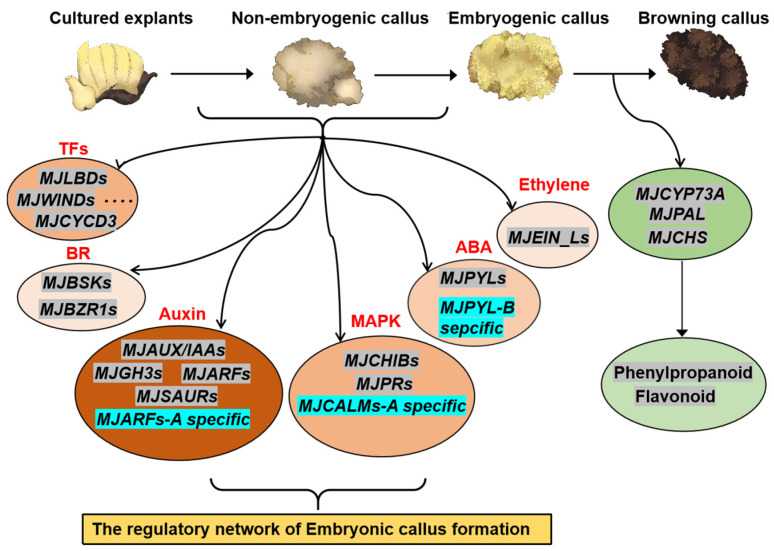
A schematic representation of gene expression regulation during the induction of embryogenic callus from explants in the banana cultivar MJ. The depth of the orange color in the oval signifies the importance of the involved signaling pathways, with darker hues indicating a higher likelihood of involvement. Genes highlighted in gray denote the presence of both A and B alleles, while those in blue indicate alleles specific to A or B. The auxin signaling pathway genes are primarily responsible for the induction of embryonic callus in bananas, with significant contributions from the MAPK signaling pathway, as well as the ABA, Ethylene, and BR pathways. Importantly, transcription factors such as LBD, WIND, and CYCD3, previously identified as key players in callus induction, are also integral to the formation of embryogenic callus in bananas, establishing a comprehensive regulatory network. Furthermore, our findings indicate that the predominant cause of browning in banana embryogenic callus is the marked upregulation of genes involved in the phenylalanine and flavonoid biosynthesis pathways, resulting in the accumulation of these compounds and potentially leading to browning.

In our study of the banana ABB genotype cultivar MJ, we focused on the dominant and specific expression of the A and B alleles. Interestingly, we found that the homologous genes of the A and B alleles in MJ exhibited very similar expression patterns in the auxin, ABA, ethylene, and MAPK signaling pathways, with no homologous genes showing dominant expression levels (Figure 8). However, we identified some A-specific genes expressed in these signaling pathways, including the specific expression of the ARF gene family in the AA genome, which negatively regulates EC induction (Figure 8). *MJEIN3* in the ethylene signaling pathway also acts as a negative regulator, whereas *MJCALM* in the MAPK signaling pathway acts as a positive regulator (Figure 8). This suggests that these specific A genes have unique effects on embryogenic calli in bananas. In the B allele, we identified only two PYL genes in the ABA signaling pathway, and only one was specifically expressed in embryogenic calli, indicating that ABA signaling molecules in B-specific genes promote EC expression to some extent (Figure 8). In addition, our analysis of embryogenic calli and browned embryogenic calli revealed that browning was primarily caused by the accumulation of phenylpropanoids and flavonoids (Figure 8). Browning can be effectively suppressed by inhibiting the synthesis of these phenolic compounds, thereby enhancing the activity of banana embryogenic calli and improving their genetic transformation.

### 3.1. Auxin Master Regulator in the Induction of EC in ABB Banana

Auxins and cytokinins are primary hormones that induce callus formation. Recent studies have highlighted auxin as a well-known inducer of lateral root formation in *Arabidopsis*. In the auxin signaling pathway, enzymes encoded by GH3 genes primarily function to conjugate auxins with amino acids, thereby regulating auxin activity and levels. This regulatory mechanism helps maintain the auxin balance during various developmental stages and environmental conditions [41]. Studies have shown that within a few hours of SE induction, IAA concentration decreases while CcGH3 levels increase, resulting in reduced auxin levels [63]. These findings suggest that endogenous auxin accumulation is crucial for altering cell fate and establishing embryogenesis. Furthermore, in our studies, during the induction of embryogenic calli in bananas, we observed significant changes in *MJGH3* gene expression. Genes such as *MJGH3.8_1*, *MJGH3.1_3*, and *MJGH3.8_5* were highly expressed at the 5M stage and during both the 12M and EC stages, whereas genes such as *MJGH3.1_5* were significantly expressed during the 12M and EC stages (Figure 4a). This indicates that auxin levels in MJ banana callus induction are regulated by multiple GH3 genes, with endogenous auxin decreasing after 5M induction, promoting EC formation through precise regulation by multiple GH3 genes, differing from previous studies.

Previous studies have indicated that nearly 86 transcription factors related to auxin synthesis and signaling are differentially expressed from the dedifferentiation process to the SE development stage. In embryogenic calli, *GH3.17*, *PIN3*, *AUX.IAA*, *YUC*, *AUX/LIKE1*, and *SAUR* show highly expressed; however, their expression decreases during embryo proliferation. However, their expression levels decline as embryo proliferation progresses [64]. We found that *MJIAAs* were also significantly upregulated after callus induction (Figure 4b), suggesting that the expression changes in IAA genes during callus induction are relatively conserved. In our study, *MJSAURs* were significantly induced at the 5M stage (Figure 4e), indicating their involvement in EC formation in bananas. Our study also found significant upregulation of *MJPIN1* at the onset of the 5M stage (Figure 6d), indicating a significant correlation between PIN1 and banana EC formation, which is consistent with previous studies.

Various members of the LBD family also referred to as the ASYMMETRIC LEAVES2-LIKE transcription factors, such as LBD16, LBD17, LBD18, and LBD29, play a role in callus formation on explants cultured on callus induction medium (CIM) by interacting with ARF7 and ARF19 [65,66]. Our results indicate that *MJLBD16* was significantly induced at the 12-month stage of callus induction (Figure 6d), suggesting that its role is also conserved in monocotyledonous bananas, potentially acting as a key transcription factor that activates the expression of downstream genes related to callus induction.

ARRs play a pivotal role in the induction of callus through cytokinin signaling. These transcription factors are activated via a multistep phosphorelay process, leading to the activation of various target genes [22]. One notable target of type-B ARRs is CYCD3, which is crucial for reinitiating the cell cycle. The expression of *CYCD3* is significantly increased within an hour of cytokinin exposure, and its overexpression can promote callus formation even without external cytokinin application [67]. Although our callus-induction medium did not include exogenous cytokinins, we observed a significant increase in *MJCYCD3* expression at the 12M stage of callus induction (Figure 6d). We did not detect significantly high expression of type-B ARRs during the callus induction stage, suggesting that MJ callus formation may be promoted by the activation of *MJCYCD3* expression through endogenous cytokinins. The AP2/ERF transcription factors, including ESR (also referred to as DORNRÖSCHEN [DRN]), ESR1, and ESR2, are potential candidates involved in cytokinin-driven callus formation. Notably, the overexpression of either ESR1 or ESR2 can trigger callus formation even without the addition of external plant hormones [68,69]. However, in bananas, we found that *MJESR1* was not expressed during the callus formation, indicating that the underlying mechanism in bananas is significantly different from that in papaya.

### 3.2. The PYL Genes Play a Specific Promotive Role in the B Genome During Banana Callus Formation

In *Ormosia henryi*, the levels of IAA, cytokinins, gibberellins, and ABA significantly increase during the development of embryogenic calli (EC). The expression patterns of DEGs involved in ABA biosynthesis and signal transduction, such as ZEP, ABA2, AAO3, CYP97A3, PYL, and ABF, are consistent with endogenous hormone levels observed during SE [70]. As receptors of ABA, PYL proteins play crucial roles in ABA-mediated seed germination, with ABI5 modulating seed germination through the feedback regulation of PYR/PYL/RCAR ABA receptor gene expression [71]. In banana tissues, the expression levels of most *PYL* genes peaked during the 5M and 12M stages and gradually decreased during the EC and BC stages (Figure 5b). However, previous studies indicated that ABA represses callus formation, highlighting its negative effects on cell proliferation. In our study, we did not observe the upregulation of *PYL* during SE in banana tissues. Notably, within the hormone signaling pathways, we identified only two B-specific genes, the *MJPYL* genes, whose expression began to increase gradually from the 5M stage, reaching the highest levels in embryogenic calli (Supplementary Table S3). ABA concentration in plants increases upon exposure to salt stress. ABA binds to its receptor proteins (PYR/PYL/RCAR), thereby transmitting the ABA signal to downstream transcription factors that regulate the expression of stress-responsive genes. These findings suggest that the *PYL* gene, a downstream receptor of ABA, is significantly upregulated during banana callus formation, indicating a potential promotive role in callus development rather than merely responding to stress signals. The specific inducible expression of the two B alleles of *MJPYL* suggests that they may play a crucial role in promoting callus formation via the ABA signaling pathway. This represents a polyploid-specific expression pattern that is significantly different from that of ordinary diploids.

### 3.3. Elucidating the Primary Browning Compounds Contributes to Mitigating Browning During the Process of EC Induction

Most studies on callus responses to browning have concentrated on physiological indicators, with limited research on the transcriptomic responses of banana calli to browning [72]. In the present study, we analyzed the genetic differences between embryogenic and browning calli in bananas. We identified DEGs that were enriched in pathways such as flavonoid biosynthesis, phenylalanine metabolism, the biosynthesis of phenylalanine, tyrosine, and tryptophan, and phenylpropanoid biosynthesis. These findings suggest that phenolic compounds, which are ubiquitous secondary metabolites in plants, serve as primary substrates for enzymatic browning. These substances can be categorized as flavonoids, phenolic acids, and tannins.

Browning results from a physiological enzymatic process where phenolic production, influenced by sugars, is essential. Preventing browning can be achieved by inhibiting flavonoid production. CHS is responsible for catalyzing the first critical step in the flavonoid biosynthesis pathway [73,74]. The expression of the CHS genes was upregulated during the browning of banana explants, which accelerated the browning process (Figure 5g). Phenolic secondary metabolites significantly influence the quality of plant-derived foods by affecting their appearance, flavor, and health-promoting properties. Various factors affect the contents of these metabolites, influencing their stability, biosynthesis, and degradation. Notably, PAL is a key enzyme in their biosynthesis, inducible by diverse stress and environmental conditions [75]. PAL and peroxidase (POD) act as catalysts for polyphenol biosynthesis, leading to wound-induced browning [76]. The PAL gene family is crucial for plant growth, development, and abiotic stress response and has been identified in various plants [77]. Browning of banana fruit during ripening results from the release of pre-existing PPO enzyme, which is synthesised very early in fruit development [78]. Genome-wide analysis of the polyphenol oxidase gene family in bananas reveals that MaPPO1 and MaPPO6 are major contributors to fruit browning during fruit ripening [79]. Phenylpropanoid compounds serve multiple functions in plant defense mechanisms, acting as both preformed and inducible barriers-physical and chemical-against infections. They also function as signaling molecules that trigger defense gene activation both locally and systemically [80]. Cinnamate 4-hydroxylase (C4H) is a crucial gene within the phenylpropanoid pathway, playing a role in the regulation of flavonoid and lignin biosynthesis in plants. Additionally, cinnamyl alcohol dehydrogenase (CAD) facilitates the final step in monolignol biosynthesis by converting cinnamyl aldehydes into alcohols, utilizing NADPH as a cofactor [81]. 4-Coumarate: coenzyme A ligase (4CL) is a critical enzyme in the phenylpropanoid biosynthetic pathway [82]. The upregulation of these genes in MJ ultimately leads to flavonoid biosynthesis and the accumulation of phenylpropanoids, with the phenolic substances responsible for enzymatic browning originating primarily from the phenylpropanoid pathway (Figure 5g). These phenolic compounds cause browning in MJ embryogenic calli. By identifying the main metabolites responsible for browning in bananas, we can effectively inhibit the production of these compounds during banana tissue culture using substances that suppress their synthesis, thereby enhancing callus induction rates and transformation efficiency. Previous studies on plant tissue cultures of *Taxus chinensis* and *Glycyrrhiza inflata* identified flavonoid compounds from the phenylpropanoid pathway as the primary contributors to browning, with myricetin and quercetin being the main substrates of the browning reaction. The inhibition of flavonoid biosynthesis can prevent browning, and the use of gibberellic acid (GA3) as an inhibitor of flavonoid biosynthesis has been shown to effectively control browning [73], further confirming the role of flavonoids in inducing browning. Research has shown that incorporating ascorbic acid into basal media can significantly reduce phenolic secretion, enhancing culture quality and boosting the survival rates of okra cotyledonary node explants (*Abelmoschus esculentus* L.) [83]. Additional research has demonstrated that activated charcoal in the culture medium can mitigate the negative effects of leached phenolics on the regeneration of *Celastrus paniculatus* and *C. orchioides* [84]. Based on these insights, we suggest that testing various combinations of GA3, ascorbic acid, activated charcoal, and other additives during callus induction in the ABB genotype MJ cultivar could inhibit embryogenic calli browning, thereby enhancing the efficiency of banana genetic transformation.

## 4. Materials and Methods

### 4.1. Callus Induction

The MJ variety of bananas (*Musa* spp. cv. Bengal, ABB Group), with a genotype of ABB, was introduced from Bangladesh and cultivated on the campus of Fujian Agriculture and Forestry University. Young male inflorescences were sourced from MJ banana plants exhibiting normal flowering patterns. They were used as explant material and initially rinsed under clean running water. The outer bracts and flowers were removed, leaving an inner inflorescence approximately 4 cm in size. These were then transferred to a laminar flow hood, where the inflorescences were rinsed three times with sterile distilled water. Following this, the inflorescences were immersed in 75% ethanol for 5 min with occasional stirring. The ethanol was discarded, and the inflorescences were rinsed 3–5 times with sterile distilled water. The inflorescence is then trimmed to approximately 1.5 cm, and immature male flowers are selected for inoculation. Eight banana cultivars were collected to establish an embryogenic callus induction system. The culture medium was prepared using MS basal medium and single distilled water, incorporating chemicals such as MS basal medium salts, 2,4-D, NAA, and IAA, as outlined in Supplementary Table S1. The concentrations of NAA and IAA were 1 mg/L in the culture medium, and the concentrations of 2,4-D in media B1 to B4 were 0.5, 1, 2, and 4 mg/L, respectively. The callus induction rate statistics were analyzed using IBM SPSS Statistics v.26.0.

### 4.2. Histological Observation of Callus

Embryogenic and non-embryogenic calli were fixed and preserved using FAA fixative, composed of 50 mL of 95% ethanol, 5 mL of glacial acetic acid, 10 mL of 37% formaldehyde, and 35 mL of double-distilled water. A vacuum was applied until bubbles appeared on the surface, maintained for 10 min, and then gradually released, allowing the tissue to settle at the bottom of the tube. Post-vacuum, the FAA fixative was replenished as necessary, and samples were stored at 4 °C overnight. Dehydration was subsequently performed by transitioning to absolute ethanol, starting with 50% ethanol, each concentration applied for 60 min, followed by an overnight treatment in absolute ethanol. Sequential treatments with 75% ethanol and 25% xylene, 50% ethanol and 50% xylene, 25% ethanol and 75% xylene, and finally 100% xylene were conducted, each for 60 min, with an overnight treatment in 100% xylene. Samples were then treated at 60 °C with 25%, 50%, and 75% paraffin–xylene solutions, each for 3–5 h, and finally with 100% paraffin for one week, changing the paraffin twice daily. For embedding, the embedding box was placed on an ice–water mixture, and melted paraffin was quickly poured into it. Once the paraffin slightly solidified at the bottom, the material was positioned in the paraffin using preheated tweezers and allowed to cool. The embedded tissue was sectioned into 8 μm slices using a microtome (LEICA, RM2145, Wetzlar, Germany), and the sections were floated on water on glass slides and incubated overnight at 37 °C. The samples were deparaffinized with 100% xylene and treated twice for 20 min each. Rehydration was performed sequentially with a 50% ethanol/xylene mixture, followed by 100%, 95%, 80%, and 70% ethanol, each for 2–5 min. The samples were stained with 1% periodic acid solution for 15–20 min, followed by Schiff reagent for 10–15 min, rinsed with running water for 5–10 min, stained with 0.5% hematoxylin for 2 min, and rinsed again with running water for 5 min. A few drops of mounting resin were applied to the slide, which was then covered with a coverslip. The morphology, internal structure, and cell division of the callus tissues at various stages were observed using a Nikon Ni-U research-grade upright microscope.

### 4.3. RNA Extraction and Library Conduction

Total RNA extraction utilized the TaKaRa MiniBEST Plant RNA Extraction kit (Takara Biomedical Technology (Beijing), Beijing, China). The integrity and quality of the RNA were assessed through 1.0% agarose gel electrophoresis and NanoDrop 2000 (Thermo Fisher Scientific, Waltham, MA, USA). RNA-Seq libraries were constructed using the NEBNext^®^ Ultra™ RNA Library Prep Kit for Illumina^®^ (New England Biolabs (NEB), Ipswich, MA, USA). Prior to sequencing on the Illumina NovaSeq platform at Novogene (Beijing, China), the concentrations of the prepared libraries were quantified using the Qubit^®^ 2.0 fluorimeter (Qubit dsDNA HS Assay Kit, Thermo Fisher Scientific, Waltham, MA, USA).

### 4.4. Differential Gene Expression Analysis and Function Enrichment Analysis

Banana explants were subjected to flower culture, resulting in the generation of callus at various developmental stages, including EC and browning EC. Explants cultured on B2 for seven days were as the beginning control (MJ_0M), and after 5 months (MJ_5M) were selected for further culture, and those collected after 12 months (MJ_12M) were also cultured. The EC (MJ_EC) and browning EC (MJ_BC) obtained were then subjected to transcriptome sequencing with three replicates for each sample. Illumina sequencing was employed to generate the sequencing data, followed by quality control filtering using Fastp software (v.0.23.2, https://github.com/OpenGene/fastp (accessed on 8 July 2023). Assembly and annotation of the *M. acuminata* (AA genome) of DH-Pahang and *Musa balbisiana* (BB genome) of DH-PKW were downloaded from NCBI as reference genomes for the analysis of transcriptome data. Comparative analysis was performed using Hisat2 software [85] (v.2.1.0, https://daehwankimlab.github.io/hisat2/download/ (accessed on 15 July 2023), and feature counts were quantified using featureCounts [86] (v.2.0.2, https://github.com/ShiLab-Bioinformatics/subread (accessed on 20 July 2023) to calculate TMM (Trimmed Mean of M-values normalized data) value. Differential expression analysis was conducted using the Trinity software (v.2.11.0, https://github.com/trinityrnaseq/trinityrnaseq/wiki/Installing-Trinity, accessed on 20 July 2023), and the DESeq2 (v.1.42.0) package in R (v.4.3.1). DEGs were identified based on log2FoldChange (|log2FoldChange| > 1) and padj (<0.05). Gene Ontology (GO) enrichment analysis and Kyoto Encyclopedia of Genes and Genomes (KEGG) pathway enrichment analysis for the DEGs were conducted using the EggNOG Database (http://eggnog5.embl.de/#/app/home (accessed on 10 November 2023) and OmicShare (https://www.omicshare.com/tools/ (accessed on 15 November 2023). The visualization of the gene expression heatmap was also performed using the OmicShare platform(https://www.omicshare.com/tools/ (accessed on 15 November 2023). The prediction of the functional roles of the DEGs utilized the nr database; details regarding its setup can be found at https://www.matrixscience.com/help/seq_db_setup_nr.html (accessed on 10 December 2023).

### 4.5. Identification of Genomic Alleles in AA and BB

The collinear genes between AA and BB genome were identified using Mcscan (https://github.com/tanghaibao/jcvi/wiki/MCscan-(Python-version) (accessed on 2 August 2024), complemented by the reciprocalBLAST_plus.pl script with identity set to 0.6 and coverage set to 0.2. The remaining genes were added to the list of contributing genes through homologous alignment, resulting in a comprehensive collinearity table for AA and BB. Genes specific to AA and BB, with no matches, were further confirmed as specific genes by aligning them to the AA and BB genomes, respectively, using the GMAP software (http://research-pub.gene.com/gmap/, accessed on 20 October 2024) [87]. Additionally, seven specific genes were selected, and their adjacent genes were detected using BLAST (https://blast.ncbi.nlm.nih.gov/Blast.cgi, accessed on 20 October 2024). The positions of these specific genes were visualized using R (v.4.3.1), illustrating whether the variations were due to segment insertions or deletions. Structural variation analysis was conducted to confirm the variant genes.

### 4.6. Quantitative Analysis

The relative expression changes of six candidate genes (*MJ_A_GH3_1*, *MJ_A_IAA30_1*, *MJ_B_LAX2*, *MJ_A_CYCD3*, *MJ_B_WIND2,* and *MJ_A_AGL15*) in banana callus at five different stages were analyzed using real-time quantitative PCR (qPCR) technology. The Banana *RPS2* gene was used as the reference gene [88]. Sangon Biotech synthesised specific primers for each transcript. Extracting RNA from the material at different periods using Tiangen’s RNAprep Pure Plant Plus Kit (Polysaccharides and Polyphenolics-rich). The extracted RNA was then reverse transcribed into cDNA using the EvoM-MLV reverse transcription premix kit (Accurate Biotechnology, Changsha, Hunan, China) according to the manufacturer’s instructions. Materials from five different developmental stages (0M, 5M, 12M, EG, BC) were cultured on fresh media for 10 days before sampling. The five samples were collected around 7 p.m. and immediately frozen in liquid nitrogen. All samples were stored at −80 °C. Once all the materials were collected, we proceeded with qPCR analysis. The RT-qPCR was performed using the TB Green Premix Ex Taq II kit (Takara Biomedical Technology (Beijing), China). The amplification program was as follows: 95 °C for 2 min, 95 °C for 5 s, 60 °C for 30 s, cycle 40, followed by a melting curve analysis from 65–95 °C. Relative transcript levels were calculated with the ΔΔCt method. The primer sequences are listed in Supplementary Table S4.

## 5. Conclusions

In this study, we performed a dynamic expression analysis of the EC induction process in the banana cultivar MJ. Our findings reveal that this process is mainly regulated by the plant hormone, MAPK, and zeatin biosynthesis pathways. Notably, while homologous genes did not exhibit dominant expression between the A and B alleles, there was a marked predominance of A-specific allele expression within these pathways, with B-specific allele expression being notably scarce during EC induction. This suggests that A-specific alleles may play a pivotal role in the induction of banana EC. Furthermore, we discovered that the browning of banana callus could be attributed to the substantial upregulation of key genes in the phenylpropanoid and flavonoid metabolic pathways, resulting in the accumulation of these metabolites. Additionally, we identified several key transcription factors known to be highly expressed during the induction of banana EC. Our results provide valuable insights into the allele-specific expression effects in banana EC induction and serve as important references for enhancing the banana embryogenic callus induction system.

## Data Availability

The transcriptome data of banana callus at various developmental stages have been uploaded to the NCBI Sequence Read Archive under the accession number PRJNA1212789.

## Supplementary Materials

The following supporting information can be downloaded at: https://www.mdpi.com/article/10.3390/plants14050761/s1. Supplementary Table S1: Effects of 2,4-D on embryonic callus induction of Banana ABB cultivar MJ. Supplementary Table S2: Annotation of specific A Alleles enriched in plant hormone signal transduction, MAPK signaling pathways and zeatin biosynthesis. Supplementary Table S3: Annotation of specific A Alleles enriched in plant hormone signal transduction, MAPK signaling pathways and zeatin biosynthesis. Supplementary Table S4: Primers for RT-PCR are used to verify gene expression levels. Supplementary Figure S1: The Pearson’s correlation analysis and Principal Component Analysis (PCA) were conducted on 15 samples. Each with three biological replicates, using the AA genome as a reference. Supplementary Figure S2: Venn diagram showing overlap and specific DEGs between four samples at different stages using BB genome as reference. 0M: immature male flower cultivated on B2 medium for one week as cultured explant; 5M: flower cultivated on B2 medium for 5 months; 10M: 10-month callus; EC:embryogenic callus. BC: Browning embryogenic callus. Supplementary Figure S3: Gene Ontology (GO) analysis of differentially expressed genes across four groups. (a) The 20 most significantly enriched GO biological process categories among DEGs in the comparison of 5M versus 0M. (b) The top 20 enriched GO pathways identified in the 12M versus 5M comparison, sorted by the number of DEGs with a *p* value ≤ 0.05. (c) The 20 most enriched GO categories among DEGs in the EC versus 12M comparison. (d) The top 20 GO pathway categories in the BC versus EC DEGs. Supplementary Figure S4: KEGG analysis of differentially expressed genes across four groups. (a) The 20 most significantly enriched GO biological process categories among DEGs in the comparison of 5M versus 0M. (b) The top 20 enriched GO pathways identified in the 12M versus 5M comparison, sorted by the number of DEGs with a *p* value ≤ 0.05. (c) The 20 most enriched GO categories among DEGs in the EC versus 12M comparison. (d) The top 20 GO pathway categories in the BC versus EC DEGs.
