# Supplementary material for: Allelic Expression Dynamics of Regulatory Factors During Embryogenic Callus Induction in ABB Banana (Musa spp. cv. Bengal, ABB Group)
Source: Plants (Basel). 2025 Mar 1;14(5):761. doi: 10.3390/plants14050761 (PMC11902074; doi:10.3390/plants14050761)
Supplement: Supplementary file 1 [file plants-14-00761-s001.zip › Supplementary figure.pdf]

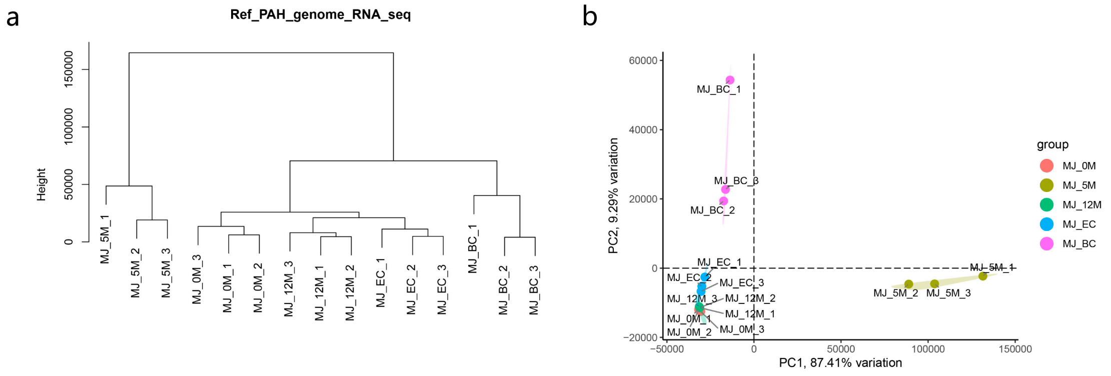

Supplementary Figure S1. The Pearson's correlation analysis and Principal Component Analysis (PCA) were conducted on 15 samples. Each with three biological replicates, using the AA genome as a reference.

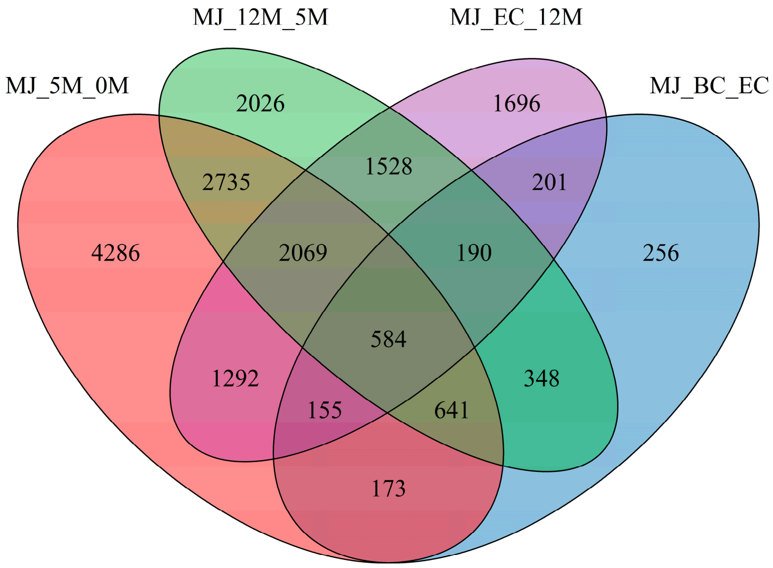

Supplementary Figure S2. Venn diagram showing overlap and specific DEGs between four samples at different stages using BB genome as reference. 0M: immature male flower cultivated on B2 medium for one week as cultured explant; 5M: flower cultivated on B2 medium for 5 months; 10M: 10-month callus; EC:embryogenic callus. BC: Browning embryogenic callus.

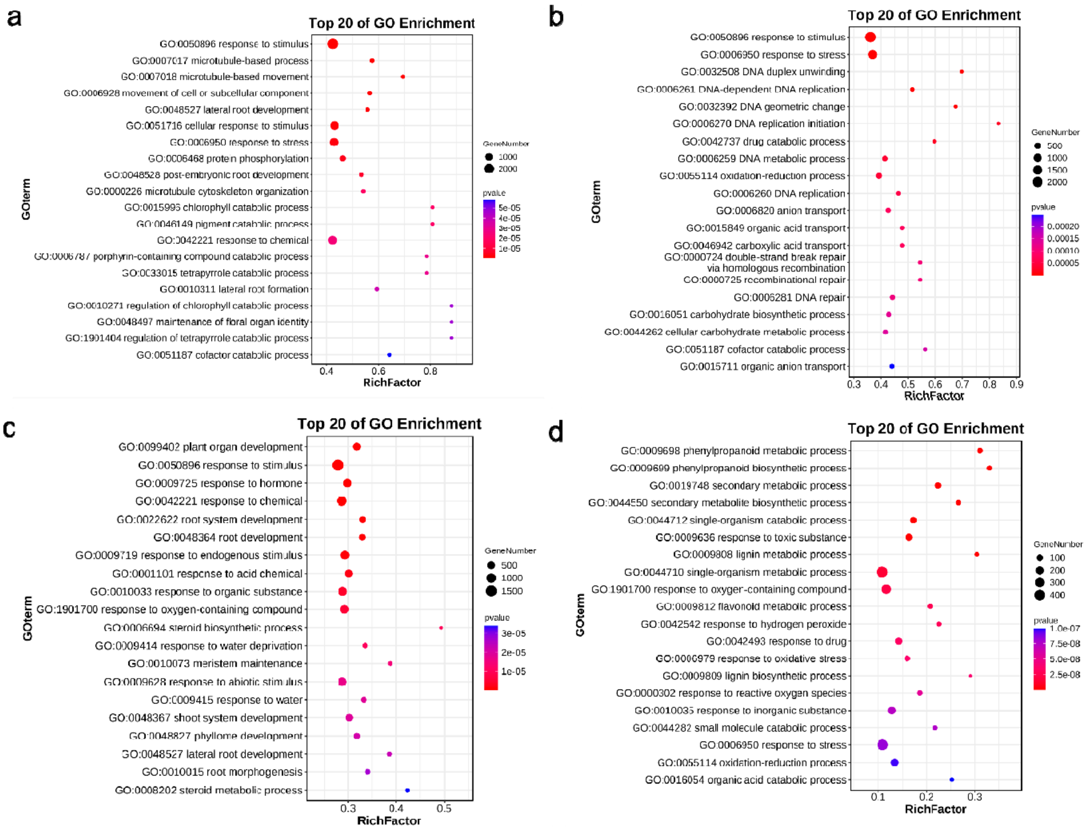

Supplementary Figure S3. **Gene Ontology (GO) analysis of differentially expressed genes across four groups. (a)**. The 20 most significantly enriched GO biological process categories among DEGs in the comparison of 5M versus 0M. (**b**). The top 20 enriched GO pathways identified in the 12M versus 5M comparison, sorted by the number of DEGs with a P value ≤ 0.05. (**c**). The 20 most enriched GO categories among DEGs in the EC versus 12M comparison. (**d**). The top 20 GO pathway categories in the BC versus EC DEGs.

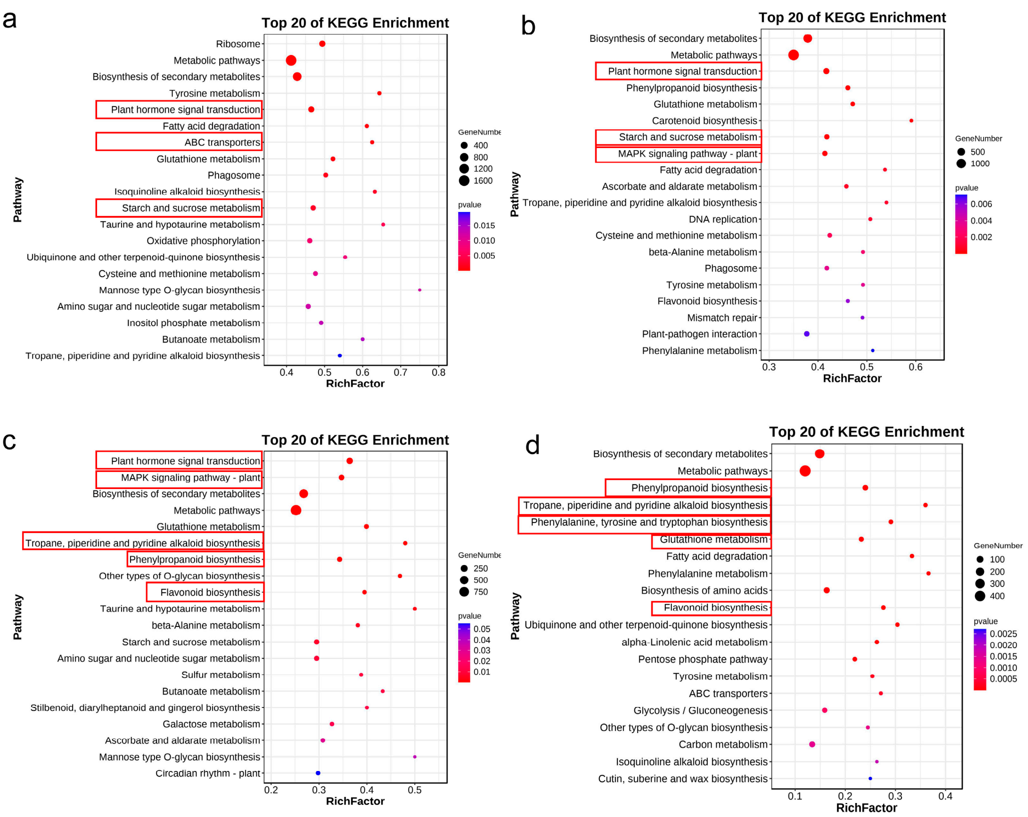

Supplementary Figure S4. **KEGG analysis of differentially expressed genes across four groups. (a)**. The 20 most significantly enriched GO biological process categories among DEGs in the comparison of 5M versus 0M. **(b)**. The top 20 enriched GO pathways identified in the 12M versus 5M comparison, sorted by the number of DEGs with a P value ≤ 0.05. **(c)**. The 20 most enriched GO categories among DEGs in the EC versus 12M comparison. **(d)**. The top 20 GO pathway categories in the BC versus EC DEGs.
